# A unified model of shared brain structural alterations in patients with different mental disorders who experience own‐thought auditory verbal hallucinations—A pilot study

**DOI:** 10.1002/brb3.1614

**Published:** 2020-04-18

**Authors:** Chuanjun Zhuo, Chunxiang Wang, Xueqin Song, Xuexin Xu, Gongying Li, Xiaodong Lin, Yong Xu, Hongjun Tian, Deguo Jiang, Wenqiang Wang, Chunhua Zhou

**Affiliations:** ^1^ Department of Biological Psychiatry School of Mental Health Jining Medical University Jining China; ^2^ The First Affiliated Hospital Zhengzhou University Zhengzhou China; ^3^ Biological Psychiatry International Joint Laboratory of Henan Zhengzhou University Zhengzhou China; ^4^ Henan Psychiatric Transformation Research Key Laboratory Zhengzhou University Zhengzhou China; ^5^ Department of Psychiatry and Neuroimaging Centre Wenzhou Seventh People's Hospital Wenzhou China; ^6^ Department of Psychiatry First Hospital First Clinical Medical College of Shanxi Medical University Taiyuan China; ^7^ MDT Center for Cognitive Impairment and Sleep Disorders First Hospital of Shanxi Medical University Taiyuan China; ^8^ Department of Psychiatric‐Neuroimging‐Genetics and Comorbidity Labotorary (PNGC_Lab) Tianjin Anding Hospital Tianjin China; ^9^ Canada and China Joint Laboratory of Biological Psychiatry Xiamen Xianye Hospital Xiamen China; ^10^ Department of Radiology MRI Center Tianjin Children Hospital Tianjin Medical University Affiliated Tianjin Children Hospital Tianjin China; ^11^ Department of Pharmacology The First Affiliated Hospital of Hebei Medical University Shijiazhuang China

**Keywords:** distinct features, own‐thought auditory verbal hallucinations, shared features, tract‐based spatial statistics, voxel‐based morphometry

## Abstract

**Objective:**

To explore shared brain structural alterations in patients diagnosed with mental disorders who experience own‐thought auditory verbal hallucinations (OTAVHs).

**Methods:**

A cohort of 143 first‐diagnosis, nonmedicated patients with OTAVHs was enrolled: 25 with schizophrenia (FUSCH‐OTAVH), 20 with major depression disorder (FUMDD‐OTAVH), 28 with bipolar disorder (FUBD‐OTAVH), 22 patients with posttraumatic stress disorder (FUPTSD‐OTAVH), 21 with anxiety disorder (FUAD‐OTAVH), and 27 with borderline personality disorder (FUBPD‐OTAVH); 25 healthy controls (HCs) participated. The Auditory Hallucinations Rating Scale (AHRS), multiple psychometric scales, voxel‐based morphometry (VBM), tract‐based spatial statistics (TBSS), and multiple regression were used.

**Results:**

Compared with HCs, patients had increased occipital cortex, dorsal prefrontal cortex (PFC), and striatum gray matter volumes (GMVs), a reduced insular cortex (IC) GMV, and an impaired frontooccipital fasciculus. The following differences were found versus HCs: FUSCH‐OTAVH, reduced PFC and occipital GMVs, increased striatum and thalamus GMVs, impaired arcuate fasciculus, u‐shaped bundle, optic tract, and upper longitudinal fasciculus (LF); FUMDD‐OTAVH, increased posterior frontotemporal junction and hippocampus GMVs; FUMN‐OTAVH, increased posterior frontotemporal junction and parietal cortex GMVs, reduced hippocampus GMV, impaired upper LF; FUPTSD‐OTAVH, increased temporal, hippocampus, and nucleus accumbens GMVs; FUBPD‐OTAVH, increased frontotemporal junction and hippocampus GMVs, impaired upper/lower LF; and FUAD‐OTAVH, increased frontal and temporal cortex, hippocampus GMVs.

**Conclusions:**

The present findings provide evidence consistent with a bottom‐up and top‐down reciprocal action dysfunction hypothesis of AVHs and with the dopamine hypothesis of AVHs. We observed specific features related to OTAVHs in patients with different mental disorders. The findings, though complex, provide clues for further studies of specific mental disorders.

## Abstract 2

We observed an unified model of shared brain structural alterations in patients with different mental disorders who experience own‐thought auditory verbal hallucinations. These findings support a unified model of own‐thought auditory verbal hallucinations.

## 1 INTRODUCTION

Auditory verbal hallucinations (AVHs)—of which there are various types, including constant commanding and commenting AVHs, replay AVHs, own‐thought AVHs (OTAVHs), and nonverbal AH—are experienced by patients with a variety of mental disorders as well as healthy individuals (Baumeister, Sedgwick, Howes, & Peters, 2017; Blom, 2015; Zhuo et al., 2019). AVHs can lead psychiatric patients to commit self‐harm, including suicide (Slotema, Bayrak, Linszen, Deen, & Sommer, 2019; Upthegrove et al., 2016). The pathological features associated with AVHs may be shared, at least in part, across distinct across neuropsychiatric diagnoses and across AVH types (International Consortium on Hallucination Research; https://ichr2017.sciencesconf.org/resource/gallery/id/2; International Consortium on Hallucination Research; www.ichr2018kyoto.jp/program.html; McCarthy‐Jones et al., 2014). Among the aforementioned AVH types, OTAVHs appear to be the most prevalent across different mental disorders (McCarthy‐Jones et al., 2014; Upthegrove et al., 2016). Based on clinical phenomenon cluster analysis findings, McCarthy‐Jones and colleagues have characterized OTAVHs as consisting of the following properties: hallucinated verbiage not addressing the person hearing them directly, first‐person voice syntax, experience similar to a memory and/or one's own inner voice or thoughts (McCarthy‐Jones et al., 2014).

Researchers conducting magnetic resonance imaging (MRI) studies aimed at exploring structural brain alterations associated with AVHs have thus far focused mostly on patients with schizophrenia (Hugdahl, 2015; Hugdahl & Sommer, 2018; Jones, 2010; Laroi et al., 2012; Waters, Woods, & Fernyhough, 2014; Zmigrod, Garrison, Carr, & Simons, 2016). Schizophrenics with AVHs have been shown to exhibit structural alterations principally in the lateral sulcus, superior temporal sulcus, and bilateral thalamus (Aleman & Larøi, 2008; Allen, Laroi, McGuire, & Aleman, 2008; Bamiou, Musiek, & Luxon, 2003; Kompus, Westerhausen, & Hugdahl, 2011; McGuire, Shah, & Murray, 1993; Modinos et al., 2013). Importantly, AVHs can be reduced in patients with schizophrenia with MRI‐guided repetitive transcranial magnetic stimulation targeting the lateral sulcus and superior temporal sulcus (Dollfus et al., 2018). Tract‐based spatial statistics (TBSS) MRI studies have revealed AVH‐associated white matter (WM) alterations in the internal capsule and anterior corona radiate of patients with schizophrenia (Di Biase et al., 2019; Xi et al., 2016; Zhang et al., 2018). There remains limited information regarding potential AVH‐related structural alterations that are similar across individuals with different diagnoses, who experience AVHs.

Here, we report a pilot study in which we used voxel‐based morphometry (VBM) (Ashburner & Friston, 2000) to assess gray matter volume (GMV) alterations and used diffusion tensor imaging (DTI) and TBSS (Bach et al., 2014) to assess WM alterations in the brains of patients with different diagnoses who experience OTAVHs. The patients enrolled in this study included first‐diagnosis, nonmedicated patients diagnosed with schizophrenia (FUSCH‐OTAVH), major depressive disorder (FUMDD‐OTAVH), mania (FUBD‐OTAVH), posttraumatic stress disorder (FUPTSD‐OTAVH), anxiety disorder (FUAD‐OTAVH), and borderline personality disorder (FUBPD‐OTAVH). Inspired by Silverstein's unified model theory (Steven, 2016), we tested the hypothesis that OTAVHs may have common neuropathological features across different mental disorders.

## 2 METHODS

### 2.1 Participants

This study was approved by the Ethics Committee at Tianjin Mental Health Centre, whose patient database was used as our source of study enrollees. Written informed consent was obtained from all participants and their legal guardians (Chinese requirement) prior to data acquisition.

The inclusion criteria for the mental disorder diagnosis groups were as follows: OTAVHs (first‐person voices, not addressing the person similar to a memory, and possibly one's own voice/thoughts (McCarthy‐Jones et al., 2014)) during MRI; mental disorder diagnosed by two senior psychiatrists according to the DSM‐IV (Tong & Phillips, 2010; Tong, Phillips, & Conner, 2016); initial diagnosis made at a mental health hospital; no pharmacological medication for at least 3 weeks before scanning; age 18–25 years; ability to comply with an MRI protocol; right‐handedness; no MRI contraindications; no history of substance abuse; and no other systemic diseases, chronic conditions, metal implants, or history of head trauma. Notably, for patients in the FUBD‐OTAVH group, first episode refers to the first episode of mania, which distinguishes bipolar disorder (a.k.a manic‐depression) from major depressive disorder (a.k.a. clinical depression).

The exclusion criteria for the patients were as follows: recurrent disorder; being enrolled in other study in the past 6 months; and comorbidity with any other neuropsychiatric disorder. The inclusion criteria for healthy controls (HCs) were as follows: no psychiatric disorders or first‐degree relatives with psychotic disorders. An intelligence quotient ≥80 was required for all participants. Cognitive ability was assessed with the Wechsler Adult Intelligence Scale, 4th edition (WAIS‐IV). Previously, the WAIS‐IV has revealed significant cognitive impairments, notably on the Verbal Comprehension Index, Cancellation, and Verbal Comprehension Index–Comprehension subtests, in schizophrenic outpatients relative to demographically matched HCs.

Applying the aforementioned criteria, we enrolled 25 FUSCH‐OTAVH, 20 FUMDD‐OTAVH, 28 FUBD‐OTAVH, 22 FUPTSD‐OTAVH, 21 FUAD‐OTAVH, and 27 FUBPD‐OTAVH patients as well as 28 HCs. We identified three healthy individuals with OTAVHs, but they were not included due to being too few in number.

### 2.2 Symptom assessment

OTAVH symptoms were assessed with the Auditory Hallucinations Rating Scale (AHRS) (Wahab et al., 2015). The MATRICS Consensus Cognitive Battery (MCCB) (Lystad et al., 2016) was used to assess cognitive ability. The Global Assessment of Functioning scale (GAF) (American Psychiatric Association, 1994) was used to assess global function. The Positive and Negative Syndrome Scale (PANSS) (Kay, Fiszbein, & Opler, 1987) was used to assess schizophrenia symptoms. The Hamilton Rating Scales for Depression (Leucht et al., 2013) and Anxiety (Hamilton, 1959) (HAMD and HAMA) were used to assess clinical depression and anxiety disorder symptoms, respectively. The Young Mania Rating Scale (YMRS) (Young, Biggs, Ziegler, & Meyer, 1978) was used to assess the manic symptoms. The Clinician‐administered PTSD Scale (CAPS) (Blake et al., 1995) was applied to assess PTSD symptoms. The sociodemographic characteristics and clinical symptoms of each group are summarized in Table 1.

**Table 1:** Sociodemographic and clinical characteristics of study participants reported as means (with standard deviations)

Variable	HC *N* = 28	SCH *N* = 25	MDD *N* = 20	AD *N* = 21	BPD *N* = 27	PTSD *N* = 22	MN *N* = 28	*F*	*p*
Age, y	25.0 (2.9)	23.2 (3.2)	24.9 (3.5)	35.0 (2.8)	20.2 (1.2)	37.9 (3.9)	16.1.9 (3.5)	93.11	<.001
Education, y	16.1 (2.5)	12.0 (2.0)	14.0 (3.5)	16.1 (3.5)	10.0 (2.5)	15.0 (3.0)	12.0 (3.5)	105.13	<.001
Illness duration, mos.	–	3.2 (1.8)	2.3 (1.9)	12.2 (1.3)	56.3 (1.9)	11.2 (1.8)	1.3 (0.5)	128.41	<.001
AHRS score	–	20.7 (5.5)	18.7 (2.3)	12.5 (2.4)	15.7 (3.0)	16.3 (2.1)	13.1 (1.5)	58.79	<.001
PANSS score	–	68.2 (1.5)	42.1 (6.8)	37.0 (2.3)	53.5 (5.9)	43.6 (9. 9)	51.2 (3.1)	127.73	<.001
HAMD score	–	32.5 5.0)	28.5 (2.4)	12.2 (3.6)	16.8 (2.2)	19.5 (3.9)	0.0 (0.0)	156.21	<.001
CAPS score	–	0.0 (0.0)	0.0 (0.0)	0.0 (0.0)	0.0 (0.0)	24.7 (2.8)	0.0 (0.0)	–	–
YMRS score	–	11.3 (2.5)	0.0 (0.0)	0.0 (0.0)	14.2 (4.5)	0.0 (0.0)	27.7 (1.4)	98.23	<.001
HAMA score	–	11.2 (1.8)	14.4 (2.1)	17.2 (3.2)	15.2 (3.6)	13.0 (3.0)	2.8 (1.2)	129.80	<.001
GAF score	98.5 (0.3)	78.0 (5.5)	80.0 (7.0)	90.2 (8.9)	76.2 (9.9)	89.2 (8.1)	80.0 (5.0)	137.9	<.001
MCCB scores									
Processing speed	46.5 (7.4)	35.1 (5.5)	39.0 (9.5)	40.0 (1.2)	38.1 (8.5)	42 (8.6)	46.0 (9.9)	101.3	<.001
Attention	46.0 (10.5)	29.3 (5.6)	24.0 (2.5)	36.0 (5.5)	28.3 (9.5)	33.0 (2.5)	26.0 (3.4)	160.1	<.001
Working memory	48.0 (10.2)	34.2 (6.4)	24.2 (10.2)	40.5 (5.4)	39.2 (9.3)	31.2 (8.2)	48.5 (9.5)	102.9	<.001
Verbal learning	48.51 (5.6)	31.4 (9.5)	34.2 (9.7)	36.5 (6.9)	37.4 (9.5)	42.2 (9.7)	48.5 (6.7)	122.7	<.001
Visual learning	43.0 (9.5)	42.0 (5.3)	35.20 (9.8)	45.2 (5.3)	42.0 (4.1)	40.2 (2.3)	40.9 (3.2)	153.1	<.001
Problem reasoning	44.7 (5.6)	36.0 (9.3)	38.0 (3.6)	42.5 (8.5)	37.0 (7.3)	40.0 (3.2)	35.5 (7.0)	111.2	<.001
Social cognition	44.5 (4.5)	35.4 (9.6)	39.4 (5.2)	40.0 (9.0)	30.4 (9.5)	37.4 (7.0)	36.9 (4.1)	179.9	<.001

Note

Patients were first‐diagnosis, nonmediated (FU) individuals who experience OTAVHs. The FU and OTAVH components of group names were omitted in observance of space limitation.

Abbreviations: mos., months; y, years.

### 2.3 MRI acquisition and image processing for VBM

Imaging data were obtained in a 3.0‐T Discovery MR750 scanner (GE, Milwaukee, WI) by three‐dimensional fast spoiled gradient echo (3D‐FSPG) recalled steady‐state acquisition with the following parameters: repetition time (TR), 10 ms; echo time (TE), 4.1 ms; inversion time, 700 ms; 10° flip angle; 24‐cm field of view (FOV); and 1.2‐mm‐thick slices (resolution, 0.47 mm × 0.47 mm × 1.2 mm). Gradient nonlinearity‐related image distortion was corrected with GradWarp, and intensity inhomogeneity was corrected with the N3 program. In preparation for automated brain‐wide VBM, 3D‐FSPGR images were subjected to bias correction, spatial normalization, tissue segmentation (gray matter, WM, and cerebrospinal fluid), and intensity modulation in SPM5 (Institute of Neurology, London, UK). The Diffeomorphic Anatomical Registration Through Exponential Lie Algebra toolbox was used for high‐dimensional normalization. For intensity modulation, voxel values from segmented images were multiplied by warped/unwarped measures derived from nonlinear spatial normalization, thereby converting relative regional gray matter density values into absolute gray matter density values (gray matter amount per unit brain tissue volume) prior to spatial normalization. Subsequently, 8‐mm Gaussian kernel smoothing of the images was completed. Age, gender, education, psychiatric symptom severity, GAF scores, and MCCB scores were treated as covariates, and covariate influences were regressed out in multiple pattern recognition analysis. The threshold for significance was family‐wise error rate corrected *p* < .05.

### 2.4 DTI

All subjects underwent DTI concurrently with 3D‐FSPGR. For DTI, a single‐shot, spin‐echo‐planar sequence with a TR of 12,000 ms and a TE of 83.3 ms was conducted to generate 4‐mm‐thick slices (no gap) with a single excitation, 26‐cm FOV, and spatial resolution of 1.02 mm × 1.02 mm × 4 mm. Diffusion properties were measured at *b* = 1,000 s/mm^2^ along 25 noncollinear directions. Eddy current and motion corrections were performed on diffusion‐weighted images with the Functional MRI of the Brain (FMRIB) Linear Image Registration Tool. Image distortion due to gradient nonlinearity image distortion was corrected for with “GradWarp.” Individual functional anisotropy (FA) and mean diffusivity (MD) maps were calculated using the DTIFIT tool.

Because of inherent MRI geometric distortion FA map differences from T1/T2/proton density‐weighted template images in SPM5, we created a FA template for the present study participants. T2‐weighted echo‐planar image was coregistered in 3D‐FSPGR images, and then, the coregistration parameter was applied to each corresponding FA map. The spatial normalization parameters from the 3D‐FSPGR images were applied to coregistered FA maps. Normalized FA maps were smoothed with an 8 mm isotropic Gaussian kernel, thereby creating averaged images (FA templates). FA maps were transformed from native space to stereotactic space by registering each image in our FA template. Finally, smoothing of the normalized FA map was completed with an 8‐mm isotropic Gaussian kernel.

### 2.5 Image processing for TBSS

Voxel‐wise statistical analysis was completed in TBSS version 1.2 software. We used the FMRIB Software Library (FSL, Oxford), including skull stripping and eddy current correction tools, to preprocess diffusion tensor images (FA, trace, axial and radial diffusivity). Briefly, FA maps created for each subject using the FSL were aligned into a common (Montreal Neurologic Institute 152 standard) space with the nonlinear registration FSL tool FNIRT. All transformed FA images were averaged to create a mean FA image, and the tracts were narrowed to generate a mean FA skeleton incorporating the central white matter tracts common to all subjects. The voxel values of each subject's FA map were projected onto the skeleton. The FA threshold was set to 0.2 (TBSS default) to confine the analysis to white matter. We conducted voxel‐wise permutation‐based nonparametric inference (Nichols & Holmes, 2002) on skeletonized the FA data using FSL Randomize version 2.1. Both patient > HC and patient < HC contrasts were identified with 5,000 permutations and a significance level of *p* < .05 (family‐wise error rate corrected). We performed multiple‐comparison corrections using threshold‐free cluster enhancement (Smith & Nichols, 2009) to avoid arbitrary selection of the cluster‐forming threshold, while preserving the sensitivity benefits of cluster wise correction. To compare trace, axial diffusivity, and radial diffusivity, we used FA images with the FSL to achieve nonlinear registration and staged skeletonization, and to estimate projection vectors from each subject onto the mean FA skeleton.

### 2.6 Statistical analyses

Sociodemographic and psychometric variables were compared among groups with LSD *t* tests. We carried out multivariate pattern recognition analysis (Elton, Chanon, & Boettiger, 2019) to assess correlations between psychometric symptoms (i.e., AHRS, PANSS, HAMD, HAMA, and YMRS, MCCB, and GAF scores) and imaging data with a significance criterion of *p* < .05.

## 3 RESULTS

### 3.1 Structural MRI analysis

**FIGURE 1:**
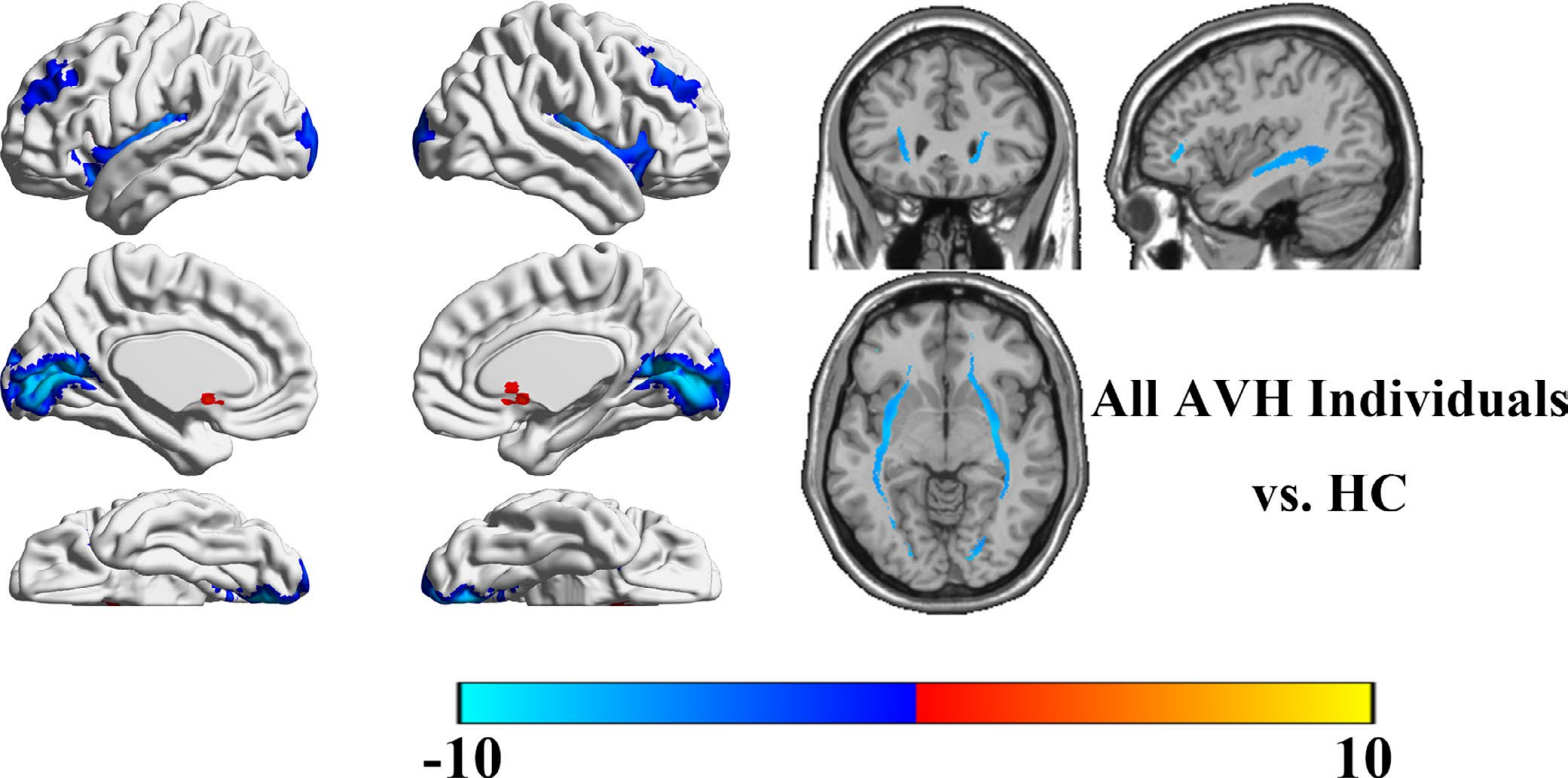
Aberrant brain structural pattern common to all examined neuropsychiatric patient groups with OTAVHs

**FIGURE 2:**
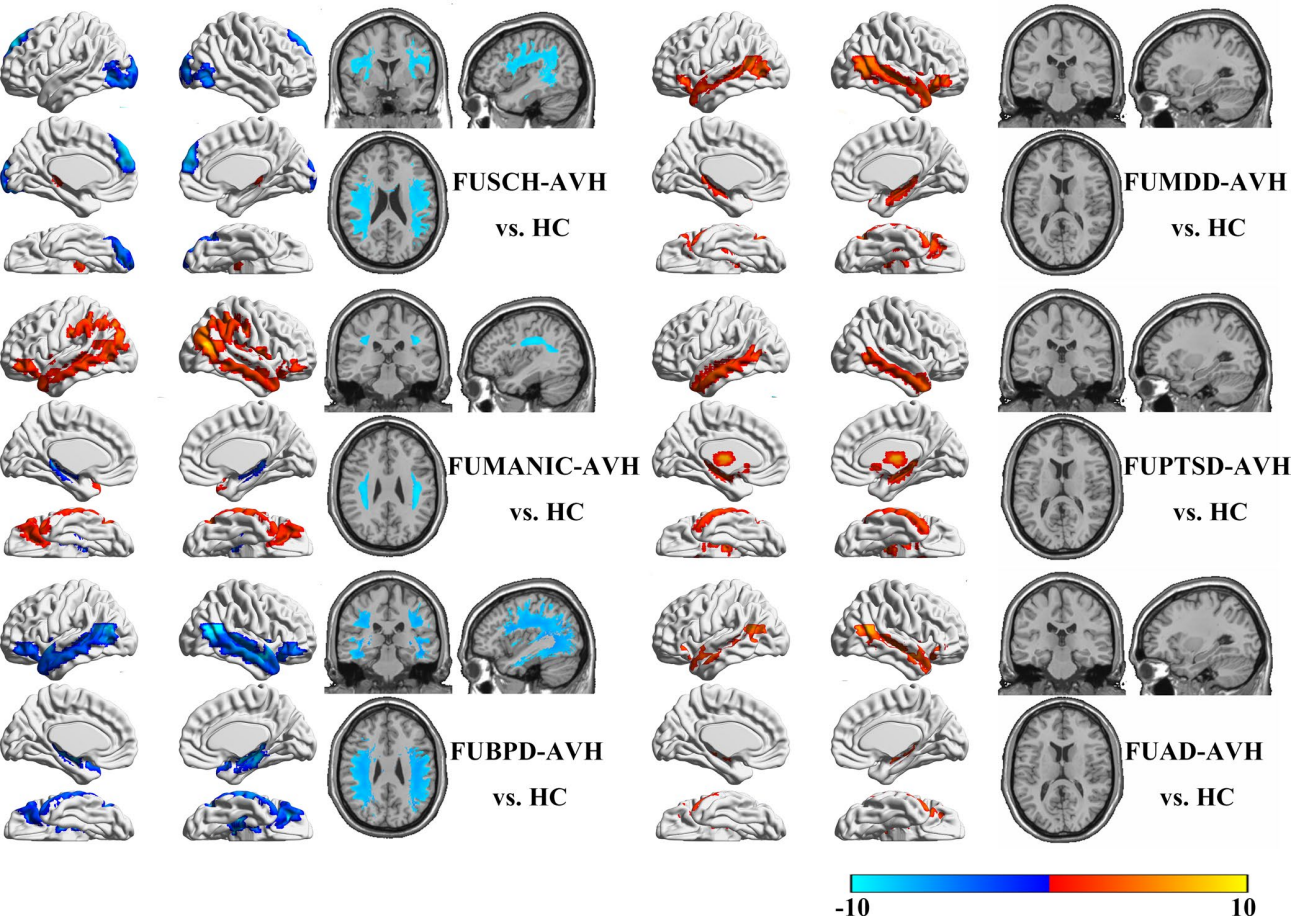
Brain structural alterations found for each neuropsychiatric patient group with OTAVHs compared with HCs

Compared with HCs, all OTAVH groups had reduced occipital cortex, dorsal prefrontal cortex (PFC), and insular cortex (IC) GMVs as well as increased striatum GMVs and impaired frontooccipital fasciculus WM tracts, as shown in Figure 1. Characteristic brain structural alterations, relative to HCs, were identified for each diagnostic group (Figure 2). Compared with HCs, the FUSCH‐OTAVH group had decreased GMVs in the prefrontal lobe and occipital cortex and had increased GMVs in the striatum and hippocampus. TBSS demonstrated decreased FA in the arcuate tract, U‐shaped fiber tract, optic tracts, and upper longitudinal fasciculus (LF). Compared with HCs, the FUMDD‐AVH group had increased GMVs in the posterior frontotemporal junction and hippocampus, without any significant WM alterations. Compared with HCs, the FUBD‐AVH group had increased GMVs in the posterior frontotemporal junction and parietal lobe, as well a reduced hippocampal GMV and decreased FA in the upper LF. Compared with HCs, the FUPTSD‐AVH group had increased GMVs in the temporal lobe, hippocampus, thalamus, and nucleus accumbens, without any significant WM alterations. Compared with HCs, the FUBPD‐AVH group had reduced GMVs in the frontal lobe, temporal lobe, and hippocampus, as well as impairments of the upper and lower LF; these alterations were notably more widespread than in the FUSCH‐AVH group. Finally, the FUAD‐AVH group had increased GMVs in the frontal lobe, temporal lobe, and hippocampus compared with HCs.

**FIGURE 3:**
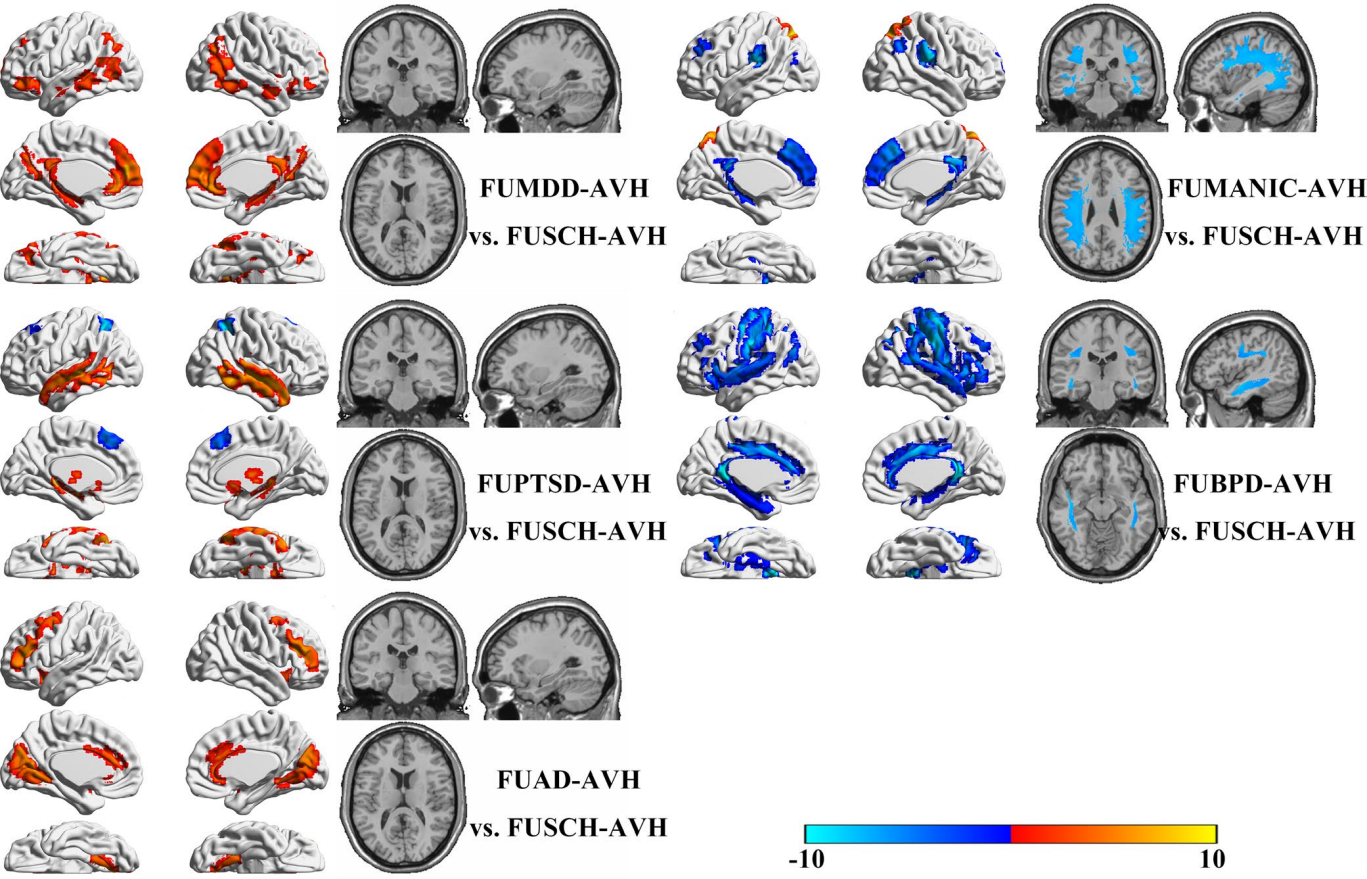
Brain structural alterations in the FUMDD‐, FUMN‐, FUPTSD‐, FUBPD‐, and FUAD‐OTAVH patient groups compared with the FUSCH‐OTAVH group

Compared with the FUSCH‐AVH group, the other five patient groups had specific characteristic differences (Figure 3). Notably, the FUMDD‐AVH group had greater GMVs in the posterior frontotemporal junction, medial PFC, anterior cingulate gyrus, posterior cingulate/precuneus, hippocampus, and bilateral inferior parietal lobule (including the angular gyrus) than were found in the FUSCH‐AVH group, without significant WM differences. Compared with the FUSCH‐AVH group, the FUBD‐AVH group had a greater GMV in the parietal lobe, but lesser GMVs in the medial PFC, cingulate gyrus, posterior parietal cortex, and hippocampus, as well as a relatively impaired upper LF, lower LF, and arcuate tract. Compared with the FUSCH‐AVH group, the FUPTSD‐AVH group had greater GMVs in the temporal lobe, hippocampus, thalamus, nucleus accumbens, and temporal lobe as well as lesser GMVs in the bilateral intraparietal sulcus, central anterior sulcus, and superior frontal sulcus (including the orbitofrontal lobe). Compared with the FUSCH‐AVH group, the FUBPD‐AVH group had widespread lesser GMVs, including in most of the frontal lobe, temporal lobe, hippocampus, anterior and posterior central gyrus, IC, and inferior parietal lobe, as well as a relatively impaired upper and lower LF. Compared with the FUSCH‐AVH group, the FUAD‐AVH group had greater GMVs in the anterior insula, anterior cingulate cortex, PFC, and occipital lobe.

**FIGURE 4:**
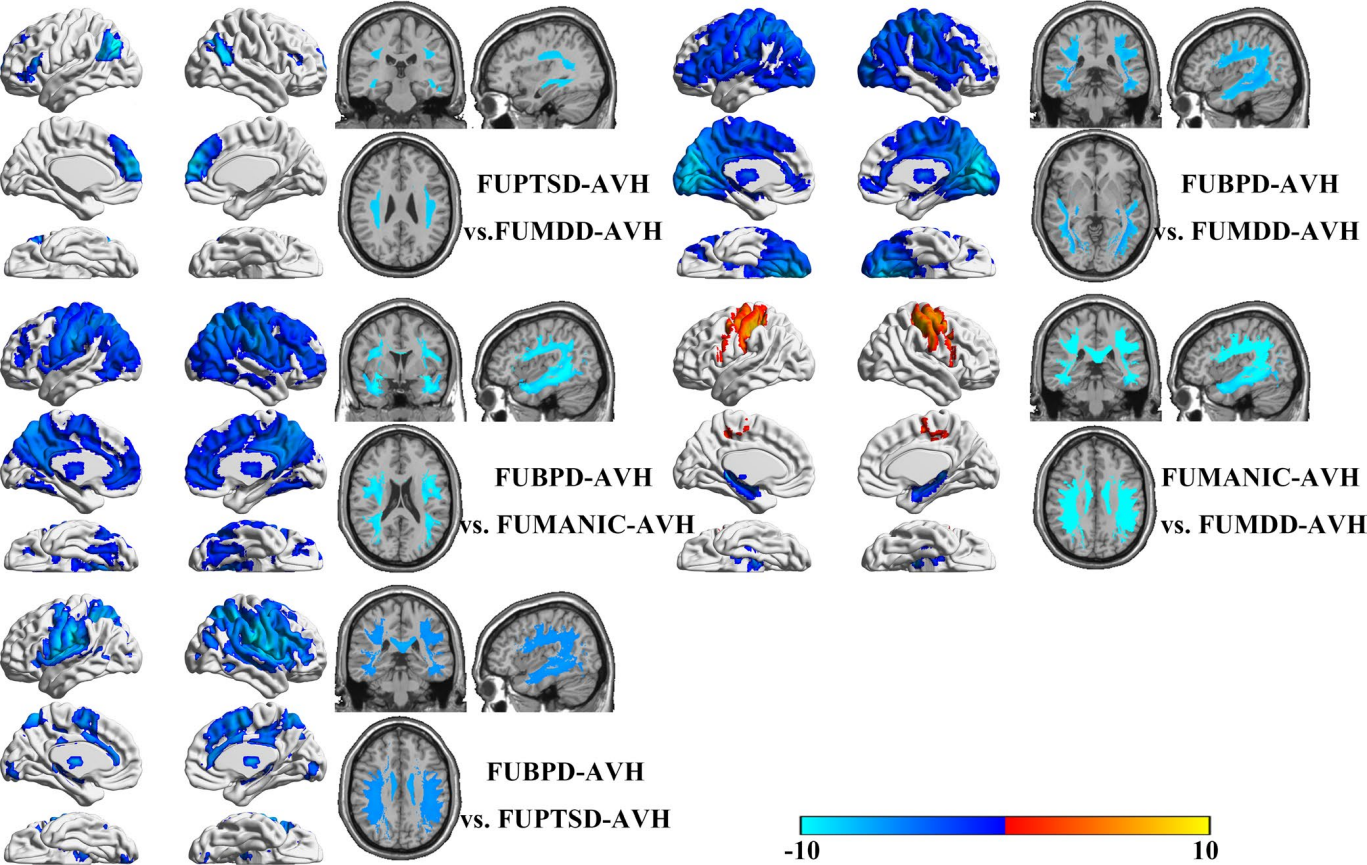
Cross comparisons of structural features among the FUBPD‐, FUPTSD‐, FUMN‐, and FUMDD groups

Comparing structural alterations among the FUBPD‐, FUPTSD‐, FUBD‐, and FUMDD‐AVH groups (Figure 4), we found that the FUPTSD‐AVH group had lesser GMVs in the medial PFC and posterior frontotemporal junction than the FUMDD‐AVH group, as well as a relatively impaired upper and lower LF. Compared with the FUBD‐AVH group, the FUBPD‐AVH group had widespread GMV reductions affecting most regions of the neocortex as well as impaired WM tracts in the upper LF, lower LF, uncinate bundle, and arcuate fasciculus. Compared with the FUPTSD‐AVH group, the FUBPD‐AVH group had widespread cortical GMV reductions and an impaired upper and lower LF. Compared with the FUMDD‐AVH group, the FUBPD‐AVH group had widespread cortical GMV reductions as well as WM tract impairments of the uncinate bundle, upper LF, lower LF, arcuate bundle, and optic nerves. Compared with the FUMDD group, the FUMN group had a greater GMV in the frontoparietal junction, lesser GMVs in the hippocampus and thalamus, and WM tract impairments of the uncinate bundle, corpus callosum, optic tracts, and the upper and lower LF (Figure 4).

### 3.2 Psychometric scores and correlation analysis

Mean AHRS, PANSS, HAMD, HAMA, CAPS, YMRS, and GAF scores and mean MCCB subscale scores obtained for each group are reported in Table 1 with the *t* and *p* values obtained for intergroup comparisons with LSD *t* tests. No GMV or FA/MD alterations were found to correlate significantly with any of the psychometrically assessed clinical symptoms.

## 4 DISCUSSION

To the best of our knowledge, this pilot study is the first study to report OTAVH‐associated brain structural alterations across a number of mental disorder diagnosis groups of patients and HCs. This work is strengthened by our having enrolled only first‐episode drug‐naive patients to avoid pharmacological influences on the brain. The present data provide three important findings for understanding the neural basis of OTAVHs, elaborated below, which are applicable to the planning of follow‐up research.

First, we observed an aberrant structural pattern common to the multiple OTAVH clinical groups examined, enabling us to identify a potential *unified model*. Based on the common structural pattern observed, we postulate that there may be common pathological features specific to OTAVH symptoms that are independent of the patients' particular mental disorder diagnosis. These findings are consistent with Hugdahl's model wherein AVHs are suggested to be a result of impairments affecting WM connections linking the temporal‐lobe auditory‐perceptual and frontal‐lobe attention‐executive networks (Hugdahl, 2015, 2017; Hugdahl & Sommer, 2018; Zmigrod et al., 2016), which are pivotal components of bottom‐up and top‐down networks, respectively. In this study, we observed reduced GMVs in the occipital cortex, dorsal PFC, and IC. According to the bottom‐up and top‐down communication disturbance hypothesis, the occipital cortex, PFC, and IC play pivotal roles in maintaining normal communication of bottom‐up and top‐down reciprocal circuitry. Structural impairments affecting these regions can thus disturb bottom‐up and top‐down reciprocal activities and thereby, perhaps, enable the development of AVH symptoms (Alonso‐Solis et al., 2015; Chang et al., 2017; Kuhn & Gallinat, 2012). The presence of impaired WM tracts linking the frontal lobe to the temporal and occipital lobes also supports this disturbance hypothesis (Catani et al., 2011; Di Biase et al., 2019; EFL, 2016; McCarthy‐Jones, Oestreich, & Whitford, 2015; Xie et al., 2019; Zhang et al., 2018). Meanwhile, the enlarged striatal GMVs in our patients are consistent with the dopamine hypothesis of AVHs (Cassidy et al., 2018; Howes & Kapur, 2009; Russo et al., 2019). The present findings thus provide evidence potentially in support of two hypotheses of AVHs while providing clues for further research into OTAVH‐specific features common to different mental disorders.

Second, we found that the FUBPD‐OTAVH patient group had more marked alterations, relative to HCs, than the other patient groups. Contrasting our FUBPD‐OTAVH and FUSCH‐OTAVH comparisons with HCs after regressing out covariates (i.e., psychometric scores and sociodemographic variables), we found, surprisingly, that the FUBPD‐OTAVH group had more widespread cortical GMV reductions and WM tract impairments. Given that there are quite limited data in the literature regarding GMV alterations and WM impairments in patients with borderline personality disorder and the available data show, for the most part, less pronounced alterations than those found in this study (Aguilar‐Ortiz et al., 2018; Gan et al., 2016; Jin et al., 2016; Maier‐Hein et al., 2014; Ninomiya et al., 2018; Rossi et al., 2015), it is difficult for us to speculate regarding an explanation for our findings in the FUBPD‐OTAVH group. The present findings may thus provide important foundational information regarding divergent neurology in patients with borderline personality disorder who experience AVHs.

Third, interestingly, we found that the FUBD‐OTAVH, FUMDD‐OTAVH, FUPTSD‐OTAVH, and FUAD‐OTAVH groups had increased GMVs in the temporal lobe, hippocampus, thalamus, frontal lobe, and parietal lobe regions compared with HCs and the FUSCH‐OTAVH group. Although similar GMV increases have been described previously in psychiatric disorder‐diagnosed patients who experience AVHs (Hugdahl, 2017; Hugdahl & Sommer, 2018; Zmigrod et al., 2016), the etiology of this phenomenon is unclear. The differences appear to be inconsistent with a functional compensation mechanism because functional compensation would not be expected to be as consistent across individuals as differences observed in this study. Further research is needed to explain these alterations.

Altogether, the present findings provide several important clues for further study: (a) impairment of components of bottom‐up and top‐down reciprocal action pathways consistent with the bottom‐up and top‐down reciprocal action dysfunction hypothesis of AVHs; (b) an association of OTAVHs with enlarged striatal GMVs, which have also been related to positive psychotic symptoms (including hallucinations), consistent with the dopamine hypothesis of AVHs; and (c) shared features among patients who experience OTAVHs with different mental disorders. Although these findings are complex and leave unresolved questions, they provide clues that are useful for guiding future research into mental disorders.

### 4.1 Limitations

This pilot study had several limitations. First, the strength of evidence was limited by the relatively small samples within each diagnostic group. We are planning future studies with larger samples to further delineate distinct neurological features of OTAVHs common to patients with different mental disorders. Second, age, gender, education level, cognitive performance, and global function differed between our study groups. Although we regressed out these factors with multiple variable pattern recognition analysis, the evidence remains weaker than could be obtained with matched samples. Third, we did not find any significant correlations between structural alterations and clinical symptoms. This lack of correlation might be due to trait features of OTAVH, but we remain cautious of accepting such an explanation given the limited amount of data available. Fourth, we focused specifically on OTAVHs. Examination of patients who experience the other types of AVHs is needed. Fifth, the accuracy of OTAVH diagnosis, which was dependent on properties of hallucinated voices reported by patients, could not be verified independently. Sixth, we compared each diagnostic study group to every other diagnostic group in this study, a statistical practice that has been questioned for potential so‐called “double dipping”. In this regard, it is difficult to balance statistical conventions that prevent type I errors versus those that enable type II errors. In the current stage of our understanding, it is important to accumulate clues and thus this potential risk is taken with due caution. Seventh, although the patient populations addressed in this study often require higher dosage pharmacotherapies than similarly diagnosed patients without OTAVHs, these findings do not provide predictive prognostic information for patients. In the future, we will conduct a follow‐up study to investigate how common, specific brain features in patients who experience OTAVHs are related to clinical implications and prognoses.

## 5 CONCLUSION

The present findings provide evidence consistent with the bottom‐up and top‐down reciprocal action dysfunction hypothesis of AVHs and the dopamine hypothesis of AVHs. We observed specific neurological features that provide clues for further research into OTAVH‐specific features common to different mental disorders and potential treatment targets for AVHs.

## CONFLICT OF INTEREST

None declared.

## AUTHORS' CONTRIBUTION

CZhuo, CW, XS, XL, and YX conceived and designed research; CZhou, XX, YX, and CW collected data and conducted research; CZhuo, HT, DJ, WW, and GL analyzed and interpreted data; CZhou and CZhuo wrote the initial paper; CZhuo, WW, and CZhuo revised the paper; WW and HT had primary responsibility for final content. All authors read and approved the final manuscript.

## Data Availability

The datasets generated and analyzed during the present study are available from the corresponding author on reasonable request.
